# Characterization of Various Subunit Combinations of ADP-Glucose Pyrophosphorylase in Duckweed (*Landoltia punctata*)

**DOI:** 10.1155/2022/5455593

**Published:** 2022-03-09

**Authors:** Mingxiu Wang, Ya Dai, Xinyu Li, Xinrong Ma, Caixia Li, Xiang Tao

**Affiliations:** ^1^College of Life Sciences, Sichuan Normal University, Chengdu, China; ^2^Chengdu Institute of Biology, Chinese Academy of Sciences, Chengdu, China; ^3^University of Chinese Academy of Sciences, Beijing, China

## Abstract

**Background:**

*Landoltia punctata* can be used as renewable and sustainable biofuel feedstock because it can quickly accumulate high starch levels. ADP-glucose pyrophosphorylase (AGPase) catalyzes the first committed step during starch biosynthesis in higher plants. The heterotetrameric structure of plant AGPases comprises pairs of large subunits (LSs) and small subunits (SSs). Although several studies have reported on the high starch accumulation capacity of duckweed, no study has explored the underlying molecular accumulation mechanisms and their linkage with AGPase. Therefore, this study focused on characterizing the roles of different *L. punctate* AGPases. *Methodology.* Expression patterns of *LpAGPs* were determined through comparative transcriptome analyses, followed by coexpressing their coding sequences in *Escherichia coli*, *Saccharomyces cerevisiae*, *Arabidopsis thaliana*, and *Nicotiana tabacum*.

**Results:**

Comparative transcriptome analyses showed that there are five AGPase subunits encoding cDNAs in *L. punctata* (*LpAGPS1*, *LpAGPS2*, *LpAGPL1*, *LpAGPL2*, and *LpAGPL3*). Nutrient starvation (distilled water treatment) significantly upregulated the expression of *LpAGPS1*, *LpAGPL2*, and *LpAGPL3*. Coexpression of *LpAGPS*s and *LpAGPL*s in *Escherichia coli* generated six heterotetramers, but only four (*LpAGPS1*/*LpAGPL3*, *LpAGPS2*/*LpAGPL1*, *LpAGPS2*/*LpAGPL2*, and *LpAGPS2*/*LpAGPL3*) exhibited AGPase activities and displayed a brownish coloration upon exposure to iodine staining. Yeast two-hybrid and bimolecular fluorescence complementation (BiFC) assays validated the interactions between LpAGPS1/LpAGPL2, LpAGPS1/LpAGPL3, LpAGPS2/LpAGPL1, LpAGPS2/LpAGPL2, and LpAGPS2/LpAGPL3. All the five LpAGPs were fusion-expressed with hGFP in *Arabidopsis* protoplasts, and their green fluorescence signals were uniformly localized in the chloroplast, indicating that they are plastid proteins.

**Conclusions:**

This study uncovered the cDNA sequences, structures, subunit interactions, expression patterns, and subcellular localization of *AGPase*. Collectively, these findings provide new insights into the molecular mechanism of fast starch accumulation in *L. punctata*.

## 1. Introduction

Duckweed is a general term for plants belonging to the *Lemnaceae* family, comprising five genera (*Spirodela*, *Landoltia*, *Lemna*, *Wolffiella*, and *Wolffia*) and more than 40 species [1]. The plants in this family do not differentiate into stems and leaves but exhibit leaflike fronds bearing none or several roots on the underside. In warm climates, duckweed thrives throughout the year, and their biomass increases significantly due to exponential growth rate in nutrient water. Indeed, duckweed can achieve between 13 to 38 metric tons dry weight (DW)/hectare/year [2, 3] depending on the environmental conditions. Duckweed can be used in bioethanol production because of its comparatively high starch and low lignin percentage [4, 5]. Through fermentation, duckweed can be used for the industrial production of biofuel and sewage treatment simultaneously [6, 7]. Also, duckweed positively affects eutrophic water because it can eliminate up to 98% of ammonia, 85% of total nitrogen, and 78% of total phosphorous [8]. Duckweed can be applied to purify foul water and generate renewable forms of energy. It is also an excellent source of animal feed [9]. Therefore, in a world of increasingly scarce resources, there is a need to constantly source for new applications of natural products.

*L. punctata* is the most efficient species in accumulating starch among over 800 *Lemnaceae* specimens collected from various parts of the world. *L. punctata* OT cultivated in wastewater increased its starch percentage to 52.9% (DW) during a field experiment [10]. In our previous study, the starch percentage of *L. punctata* 0202 plants increased from 3% to 45.5% (DW) when shifted from standard Hoagland nutrient solution to distilled water [11]. Additionally, the enzymatic activity of ADP-glucose pyrophosphorylase (AGPase, EC: 2.7.7.27) increased by 53.1%.

As one of the essential enzymes in starch biosynthesis, AGPase is the first and most crucial rate-limiting enzyme and thus has been a genetic engineering target for crop improvement [12]. Regulating AGPase enzyme activity can change the carbon metabolism of plants and influence their starch content [13]. In higher plants, AGPase functions as a heterotetramer comprising two large subunits (LSs) and two small subunits (SSs) [14, 15], whereas, in cyanobacteria and prokaryotes, it functions as a homotetramer [16]. Generally, the primary function of the large AGPase subunit is to modulate the regulatory properties of the enzyme in higher plants, while the small subunit mainly functions in catalysis [17]. However, the small subunit exhibited partial regulatory activity, and the large subunit had a little catalytic activity [18–20].

In specific instances, starch-derived and reservoir organs of plants harbor different AGPase large subunits. The expression patterns of AGPases vary in different organs and can also differ in the same organ at different developmental stages [21]. This spatiotemporal specific expression enables various AGPase to regulate starch synthesis in different organs at different plant developmental stages. AGPase is located in the plastids of most plant tissues, while most of the AGPase in rice [22], maize [23], barley endosperm [24], and early-stage wheat endosperm [25] are located in the cytoplasm. Thus, plants have both cytosolic AGPase and plastidial AGPase. To understand the role of different AGPases in plant starch synthesis, the enzymatic activity and spatiotemporal specificity of various subunit combinations need to be studied. Among the five *Lemnaceae* genera, *Spirodela* and *Lemna* are the most studied. However, the value of *Landoltia* has not been comprehensively explored, and its genome has not been decoded. Additionally, the relationship between various AGPases and starch accumulation has not been elucidated in *Landoltia*.

Our previous study demonstrated that some transcripts encoding AGPase and granule-bound starch synthase (GBSS) were upregulated, while some transcripts encoding enzymes involved in starch consumption were downregulated [11]. There are two small and three large AGPase subunit encoding genes in the *Landoltia* transcriptome; however, it remains unrevealed which large/small subunit combinations play a major role in starch accumulation. In this study, five cDNAs encoding AGPase subunits were cloned. Yeast two-hybrid (Y2H) experiments and bimolecular fluorescence complementation (BiFC) analysis were performed to study the relationship between small and large AGPase subunits. The bacterial coexpression technique was used to analyze the enzymatic activities of different large/small subunit combinations to determine which subunit combination could form AGPase with high enzyme activity. This study lays the foundation for further studies on the structure and function of AGPase, elucidating the regulation mechanism of starch synthesis and developing duckweed as renewable bioenergy.

## 2. Materials and methods

### 2.1. Plant Material and Growth Conditions

This study used *Landoltia punctata* 0202, a local duckweed strain, as the plant material. The duckweed was originally collected from Sichuan by the Chengdu Institute of Biology, Chinese Academy and Sciences. The plants were cultivated in a standard Hoagland nutrient solution under a 16 h light/8 h dark cycle, with a light intensity of 130 *μ*mol/m^2^/s at 24°C.

### 2.2. cDNAs Cloning of *LpAGPs*

Primers used for cloning were designed according to the assembled transcript sequences [11] using Primer Premier 5.0 software (Premier Biosoft International, Palo Alto, CA, USA). The primers also included restriction site oligonucleotides (Table S1) to enable further cloning. Total RNA of *L. punctata* was extracted from 200 mg fronds using OMEGA™ Plant DNA/RNA Kit (OMEGA, New York, NY, USA) according to the manufacturer's instructions. Genomic DNA was digested using Recombinant DNase I (RNase-free) (Fermentas, Waltham, MA, USA). Subsequently, total RNA was reverse transcribed into first-strand cDNA by Reverse Transcriptase M-MLV (RNase H-) (Fermentas, Waltham, MA, USA) using oligo (dT) as primers. PCR amplification was performed using KOD-Plus-Neo DNA polymerase (ToYoBo, Tokyo, Japan). The PCR products were separated by agarose gel electrophoresis and purified using EasyPure® Quick Gel Extraction Kit (TransGen Biotech, Beijing, China).

### 2.3. Yeast Two-Hybrid Analysis

Primers were designed based on the *LpAGP* sequences without the regions encoding the plastid transit peptides (Table S1). The purified PCR products of *LpAGPS*s and pGBKT7 yeast expression vectors (Clontech) were double-digested with *Nco* I/*Bam*H I (TaKaRa, Shiga, Japan) and separated by agarose gel electrophoresis. Next, the desired bands were extracted from the gel and purified using EasyPure® Quick Gel Extraction Kit (TransGen Biotech, Beijing, China). The purified products were then ligated by T4 DNA ligase (TaKaRa, Shiga, Japan) to obtain recombinant plasmids pGBKT7-*LpAGPS1* and pGBKT7-*LpAGPS2*. The purified PCR products of *LpAGPLs* were digested with *Pci* I/*Eco*R I (TaKaRa, Shiga, Japan), while pGADT7-Rec was double-digested with *Nco* I/*Eco*R I (TaKaRa, Shiga, Japan). After purification, the digestion fragments of *LpAGPLs* and pGADT7-Rec were ligated by T4 DNA ligase (TaKaRa, Shiga, Japan) after purification and transformed into *E. coli* DH5*α* competent cells via electroporation. Positive clones were identified by colony PCR, and the recombinant plasmids pGADT7-Rec-*LpAGPL1*, pGADT7-Rec-*LpAGPL2*, and pGADT7-Rec-*LpAGPL3* were obtained. The *LpAGPS* and *LpAGPL* recombinant plasmids were cotransformed pairwise into *Saccharomyces cerevisiae* strain AH109 and used for a two-hybrid analysis as per the Clontech protocol. Meanwhile, pGADT7 and pGBKT7-*LpAGPS1* were cotransformed into *S. cerevisiae* strain AH109 as the negative control, while the same strain was used to cotransform pGADT7-T and pGBKT7-53 (Clontech, Tokyo, Japan) as a positive control. AH109 transformants were selected using a synthetic growth medium lacking His, Leu, and Trp. The selection of subunit interactions was confirmed on SD/-Leu-Trp-His-Ade and X-*α*-gal medium.

### 2.4. Coexpression of AGPase LSs and SSs in *E. coli*

Primers were designed based on *LpAGP* sequences without plastid transit peptide coding region (Table S1). The PCR products of *LpAGPSs* and pACYC-Duet-1 were purified and double-digested using *Nco* I/*Bam*H I (TaKaRa, Shiga, Japan), then ligated. Meanwhile, the PCR products of *LpAGPLs* and pRSF-Duet-1 were purified and double digested using *Hind* III/*EcoR* I (TaKaRa, Shiga, Japan), then ligated. The products were transformed into *E. coli* DH5*α* competent cells, and positive clones were identified by colony PCR. Finally, the plasmids were extracted and sequenced by the Sanger sequencing method (ABI 3730 DNA sequencer, Life Technologies, USA).

The identified recombinant plasmids of *LpAGPS*s (pACYC-*LpAGPS1* and pACYC-*LpAGPS2*) and *LpAGPL*s (pRSF-*LpAGPL1*, pRSF-*LpAGPL2*, and pRSF-*LpAGPL3*) were cotransformed pairwise into *E. coli* BL21 (DE3). Meanwhile, plasmids *LpAGPS1*-pMD18-T, *LpAGPS2*-pMD18-T, *LpAGPL1*-pMD18-T, *LpAGPL2*-pMD18-T, *LpAGPL3*-pMD18-T, pACYCDuet-1, and pRSFDuet-1 were separately transformed into *E. coli* BL21 (DE3) as a negative control. Bacterial colonies containing one or two expression plasmids were selected using antibiotics. Positive clones were identified by colony PCR, and the positive *E. coli* strains were grown in Luria-Bertani medium supplemented with corresponding antibiotics at 37°C and 220 rpm overnight until an OD600 of 0.9 was achieved. After that, 1 mM isopropyl *β*-D-thiogalactoside (IPTG) was added, and the bacterial culture was further incubated at 37°C and 220 rpm for 30 h, to induce the expression of *LpAGP*s. The cultures were then centrifuged, washed, and resuspended in distilled water to OD600 of 0.8-1.0, suitable for the glycogen accumulation assay [16]. The experimental procedure was slightly modified: after adding 300 *μ*L iodine solution (0.01 M I_2_ and 0.03 M KI) to 500 *μ*L of cell suspensions, the solution was mixed and incubated at room temperature for 2-3 min. Glycogen accumulation in the cells was indicated by brownish coloration, and the different shades of the color indicated different amounts of accumulated glycogen.

### 2.5. Subcellular Localization

The PCR products of *LpAGPs* and 16318-hGFP obtained from the yeast expression vectors were purified, double-digested using *Sal* I/*Bam*H I (TaKaRa, Shiga, Japan), and then ligated. Positive clones were verified by colony PCR, restriction analysis, and sequencing, then transformed into *E. coli* DH5*α*. The endotoxin plasmids were identified by colony PCR and then extracted; the concentration was measured using NanoDrop. Healthy *A. thaliana* plants (3-4 weeks old) were selected, and 5-7 true leaves were used to isolate protoplasts according to the protocol described previously [26].

Leaf blades were cut into filaments (0.5-1 mm wide) and placed in 0.4 M mannitol. The filaments were completely immersed in the enzyme solution and incubated on a rotary shaker (40 rpm) in the dark for 2-3 h. Next, W5 solution was added to terminate enzymatic hydrolysis. The hydrolysate was sucked up with a 50 mL syringe (without needle), filtered into a round-bottom centrifuge tube through a 450 *μ*m nylon mesh, and then centrifuged at 4°C, 100 g for 8 min with acceleration and deceleration set at 3-4 gear. Afterward, the supernatant was discarded, and the protoplasts were suspended in precooled W5 solution and then centrifuged at 4°C, 100 g, for 8 min. Next, the supernatant was removed, and the protoplasts were resuspended and incubated on ice for 30 min. Subsequently, the supernatant was removed, and the protoplasts were resuspended in MMG solution (the final number of protoplasts was about 2 × 10^5^ mL^−1^).

Exactly 10 *μ*L plasmid was added to 200 *μ*L of protoplast in 2 mL tube and incubated for 5 min. Then, 210 *μ*L PEG solution was added to the mixture, mixed gently, and incubated at 23°C for 5-30 min. Subsequently, 800 *μ*L W5 solution was added to the mixture, mixed gently, and then centrifuged at 23°C, 100 g for 8 min. The supernatant was then gently discarded, and the pellet was resuspended in 1 mL WI solution, incubated for 12 h at 20-23°C under weak light, and then centrifuged at 23°C, 100 g for 8 min. After that, the supernatant was eliminated, 500 *μ*L WI solution was added, and the mixture was incubated for 20 min in the dark, then centrifuged at 23°C, 100 g for 8 min. The supernatant was again discarded, and 1 mL WI solution was added; then, the mixture was centrifuged at 23°C, 100 g, for 8 min. Thereafter, 500 *μ*L WI solution supplemented with 1 *μ*L 4′,6-diamidino-2-phenylindole (DAPI) was added after discarding the supernatant, and the mixture was agitated, followed by incubation for 5 min in the dark, and centrifugation at 23°C, 100 g, for 8 min. Finally, the supernatant was discarded, and 1 mL WI solution was added; then, the mixture was agitated, incubated for 5 min in the dark, and centrifuged at 23°C, 100 g, for 8 min. Fluorescence images were taken under excitation light of 405 nm and 488 nm using a confocal microscope.

### 2.6. Bimolecular Fluorescence Complementation (BiFC)

Primers were designed based on the *LpAGP* sequences without the regions encoding the plastid transit peptide (Table S1). About 5 mL YEB culture medium supplemented with appropriate selection antibiotics (rifampicin + kanamycin) was inoculated with *Agrobacterium* strain GV3101 that had previously been transformed with fusion protein binary vector. The culture was left to grow overnight at 28°C and 200 rpm. About six weeks old and healthy *Nicotiana tabacum* plants were prepared for BiFC analysis. First, the regions of the leaves were prepared for infiltration by puncturing them with a thin needle. Next, the syringe tip (without needle) was placed against the upper side of the leaf and pressed down slowly on the plunger, while the upper side of the leaf was supported with a finger. The infiltrations were repeated in the two different midrib regions of three leaves per plant. The infiltrated regions were marked in black for further identification.

The infiltrated plants were placed in a growth cabinet under normal growth conditions for 72 h. Afterward, 1-2 cm^2^ of the leaf segments within the infiltrated zone was excised and mounted on glass microscope slides containing water droplets. The slides were coved with coverslips and examined for expression using a confocal or epifluorescence microscope.

## 3. Results

### 3.1. Cloning and Analysis of *L. punctata* AGPase Subunit cDNAs

**Figure 1:**
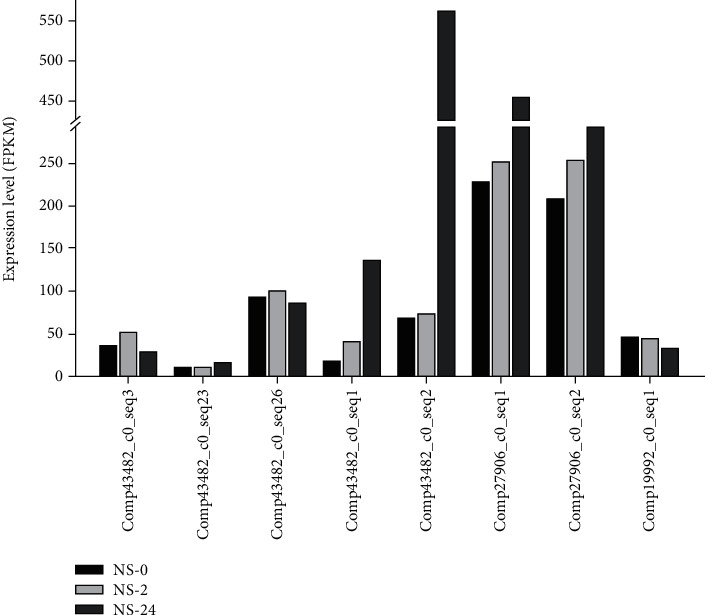
Expression patterns of *LpAGPs* during starch accumulation under nutrient starvation. *Landoltia punctata* 0202 was cultivated in standard Hoagland nutrient solution for 14 days, and then 0.5 g fronds were transferred into 50 mL distilled water in 250 mL culture flask for further cultivation. Frond samples were collected at 0 h (NS_0), 2 h (NS_2), and 24 h (NS_24) for RNA-Seq. NS: nutrient starvation; comp27906_c1_seq1: *LpAGPS1*; comp27906_c1_seq2: *LpAGPS1*; Comp19992_c0_seq1: *LpAGPS2*; comp43482_c0_seq3: *LpAGPL1*; comp43482_c0_seq23: *LpAGPL1*; comp43482_c0_seq26: *LpAGPL1*; comp43464_c0_seq1: *LpAGPL2*; comp43482_c0_seq2: *LpAGPL3*.

Comparative transcriptome analysis was performed in a previous study to investigate the high starch accumulation of *L. punctata* under nutrient starvation (distilled water treatment) [11]. It was shown that AGPase large subunits (LSs) are encoded by three genes (*LpAGPL1*, *LpAGPL2*, and *LpAGPL3*), while small subunits (SSs) are encoded by two genes (*LpAGPS1*, *LpAGPS2*) [11]. The expression levels analysis of the five *LpAGPs* showed that *LpAGPS1* (comp27906_c1_seq1, comp27906_c1_seq2), *LpAGPL2* (comp43464_c0_seq1), and *LpAGPL3* (comp43482_c0_seq2) were significantly upregulated under nutrient starvation (distilled water treatment) during the first 24 h (Figure 1). The expression patterns of the five *LpAGP*s have been verified by qRT-PCR in previous study [27]. Comp27906_c1_seq1 and comp27906_c1_seq2 possess an identical open reading frame (ORF) (*LpAGPS1*), whereas comp43482_c0_seq3, comp43482_c0_seq23, and comp43482_c0_seq26 possess an identical *LpAGPL1* ORF.

Five *LpAGPs* were cloned according to their ORFs. The length of these *LpAGPs* genes ranges from 1254 bp to 1611 bp, corresponding to 417 to 536 amino acids. Sequence comparison of the five *LpAGP* revealed that *LpAGPL2* and *LpAGPL3* share the highest sequence identity (67%), while *LpAGPS2* shares the lowest sequence identity with the other *LpAGPs*. These results show that the large subunit of *L. punctata* is relatively conserved, and the similarity between small subunits is lower than between large subunits. The sequences of *LpAGPs* were also aligned using BLASTN against the GenBank database. The gene encoding mannose-1-phosphate guanyltransferase in *Hordeum vulgare* shares significant sequence homology with *LpAGPS1* (XM_045126568.1, sequence identity of 77%). Moreover, sequence identity between *LpAGPS2* and the gene encoding the small subunit of *Colocasia esculenta* AGPase (MT445784.1) is 82%. The *LpAGPLs* show remarkable sequence homology (sequence identity ranges from 85% to 89%) to *S. polyrhiza* (JN180634.1, JN180635.1, JN180636.1).

**Figure 2:**
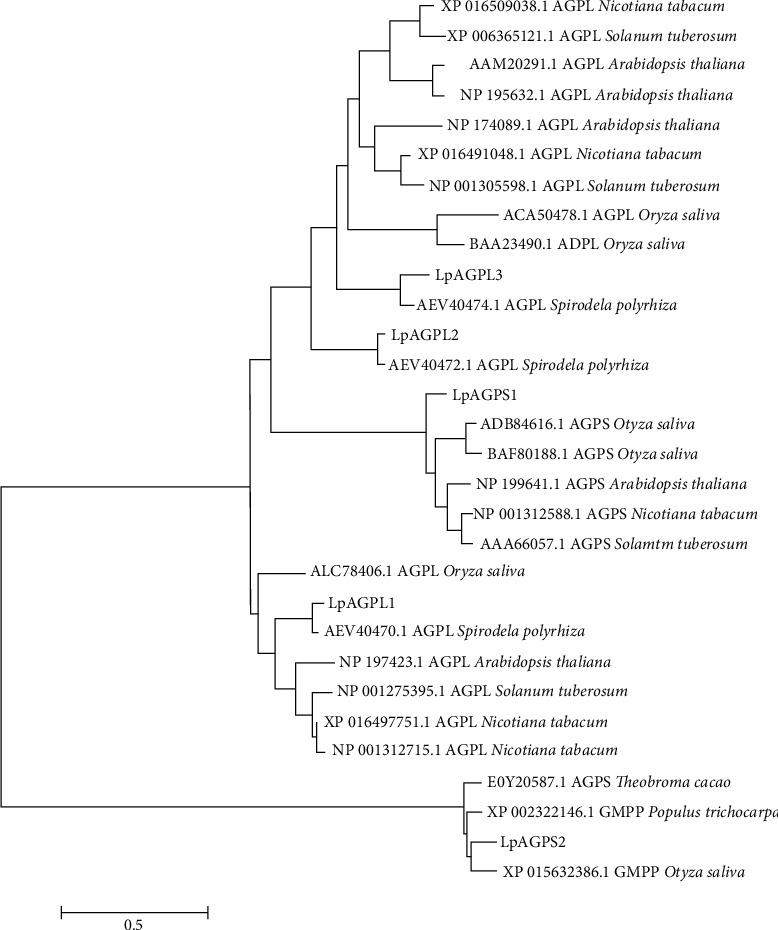
Phylogenetic analyses of AGPase large and small subunits between *L. punctata* and other plants. Protein sequences were retrieved from NCBI, and the plastid transit peptides were cut off. Then, protein sequence alignment and calculation of phylogenetic distance were performed by ClustalW. The phylogenetic tree was generated by MEGA 6.0.

A phylogenetic tree was constructed using MEGA 6.0 (Figure 2) based on the deduced amino acid sequences of the five *LpAGPs* and AGPase subunits from other plant species. Sequences alignment was conducted using ClustalW2, and the phylogenetic parameters were estimated via maximum likelihood analyses. The phylogenetic tree was created using MEGA 6.0 (Figure 2). Data were obtained from the National Center for Biotechnology Information (NCBI) (https://www.ncbi.nlm.nih.gov/) database. The results revealed that LpAGPS2 is less related to the other four LpAGPs but is more closely related to AGP of *Oryza sativa*, *P. trichocarpa*, and *T. cacao*. LpAGPS1 is more related to *O. sativa*, *A. thaliana*, *N. tabacum*, and *Solanum tuberosum*. The three AGPase large subunits of *L. punctata* were further aligned with those of *S. polyrhiza*. The sequence identity between LpAGPL1 and SpAGPL1 (AEV40471) is 94.39%, LpAGPL2 and SpAGPL2 (AEV40472) is 95.52%, and LpAGPL3 and SpAGPL3 (AEV40474) is 83.30% (Table 1). These results suggest that AGPase LSs of *L. punctata* and *S. polyrhiza* may have similar functions.

### 3.2. Interactions between the Large and Small Subunits (LSs and SSs) of *L. punctate* AGPase (LpAGP)

#### 3.2.1. Yeast Two-Hybrid and X-*α*-gal Assay

**Figure 3:**
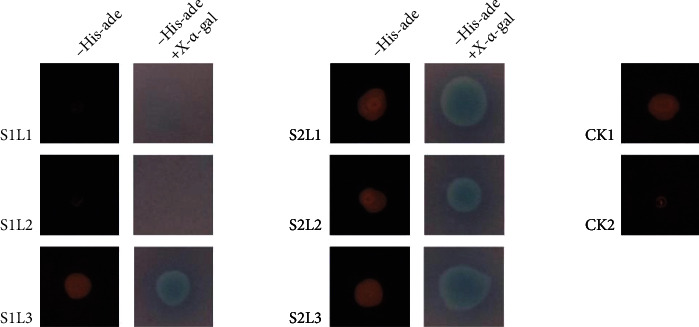
Yeast two-hybrid analyses of *L. punctata* AGPase subunit interactions. The yeast two-gybrid analyses were carried out on a SD/-Leu-Trp-His-Ade medium and confirmed on a SD/-Leu-Trp-His -Ade + X-*α*-gal medium. CK1: positive control, pGADT7-T/pGBKT7-53; CK2: negative control, pGADT7, and pGBKT7-*LpAGPS1*; S1: LpAGPS1; S2: LpAGPS2; L1: LpAGPL1; L2: LpAGPL2; L3: LpAGPL3.

Yeast two-hybrid assay (Y2H) was performed to determine the interaction between the small and large LpAGP subunits. Five *LpAGP*s were cloned and inserted into pGBKT7 (*LpAGPS1*, *LpAGPS2*) or pGADT7-Rec (*LpAGPL1*, *LpAGPL2*, *LpAGPL3*) vectors to construct recombinant plasmids. *LpAGPSs* recombinant plasmids were free combined with those of *LpAGPLs* and cotransformed into *S. cerevisiae* strain AH109 for Y2H assay. The yeast cells harboring the S1-L1 combination and the other five SS-LS combinations grew on both the selective-interaction medium (a synthetic growth medium lacking Trp, Leu, Ade, and His) and X-*α*-gal medium (Figure 3). These results indicate that LpAGPS1 does not interact with LpAGPL1.

#### 3.2.2. Bimolecular Fluorescence Complementation

**Figure 4:**
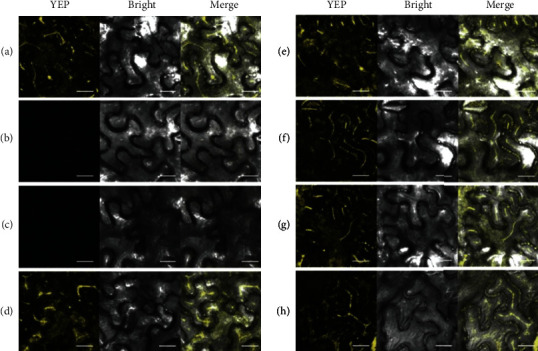
The results of bimolecular fluorescence complementation (BIFC) assay. Scale bar, 20 *μ*m. (a) Positive control (pSPYNE-ABI2 and pSPYCE-RCAR1). (b) Negative control (pSPYNE-*LpAGPS2*). (c) LpAGPS1-LpAGPL1. (d) LpAGPS1-LpAGPL2. (e) LpAGPS1 -LpAGPL3. (f) LpAGPS2-LpAGPL1. (g) LpAGPS2-LpAGPL2. (h) LpAGPS2-LpAGPL3.

Five *LpAGP*s were subcloned into the pSPYNE-35S BiFC (bimolecular fluorescence complementation) expression vector and then transferred into *A. tumefaciens* GV3101 competent cells via electroporation. Positive control (pSPYNE-ABI2 and pSPYCE-RCAR1) exhibited relatively high transient expression of YFP (Figure 4(a)), while no fluorescence was observed in the negative control (pSPYNE-*LpAGPS2*, Figure 4(b)). Besides, no fluorescent signal was observed under simultaneous expression of *LpAGPS1* and *LpAGPL1*. Simultaneous transient expressions of *LpAGPS1* and *LpAGPL3*, *LpAGPS2* and *LpAGPL1*, *LpAGPS2* and *LpAGPL2*, and *LpAGPS2* and *LpAGPL3* in *Arabidopsis* protoplasts resulted in stronger BiFC fluorescence than *LpAGPS1* and *LpAGPL2* expression. These results confirm that the LpAGPS1 does not interact with LpAGPL1.

#### 3.2.3. Coexpression of *L. punctata* AGPase LS and SS cDNA in *E. coli*

**Figure 5:**
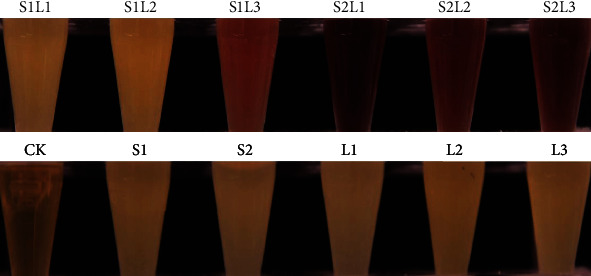
Enzyme activity analyses between large and small subunit combinations of LpAGPs. CK: control check, pRSF, and pACYC; S1: pACYC-*LpAGPS1*, S2: pACYC-*LpAGPS2*, L1: pRSF-*LpAGPL1*, L2: pRSF-*LpAGPL2*, L3: pRSF-*LpAGPL3*.

Previous studies have shown that coexpressing of cDNA sequences of plant AGPase LSs and SSs in *E. coli* yields functional heterotetrameric enzymes, as detected by iodine staining of glycogen for the product of AGPase [28]. In this study, the coding regions of *LpAGPS* and *LpAGPL* were cloned into the expression vectors pACYC-Duet-1 and pRSFDuet-1, respectively. Subsequently, the recombinant plasmids were transformed alone or pairwise into *E. coli* strain BL21 (DE3) and analyzed by iodine staining. The *E. coli* BL21 (DE3) expressing individual subunits showed no visible brownish color, indicating that single subunits cannot produce glycogen. Meanwhile, simultaneous expression of *LpAGPS1*/*LpAGPL3*, *LpAGPS2*/*LpAGPL1*, *LpAGPS2/LpAGPL2*, and *LpAGPS2*/*LpAGPL3* formed functional enzyme with cells displaying a brownish color when exposed to iodine staining (Figure 5). Because the color depth of the stained cells was determined by glycogen accumulation, indicating AGPase activities, it can be deduced that LpAGPS2 may have higher regulative activity than LpAGPS1.

#### 3.2.4. Subcellular Localization

**Figure 6:**
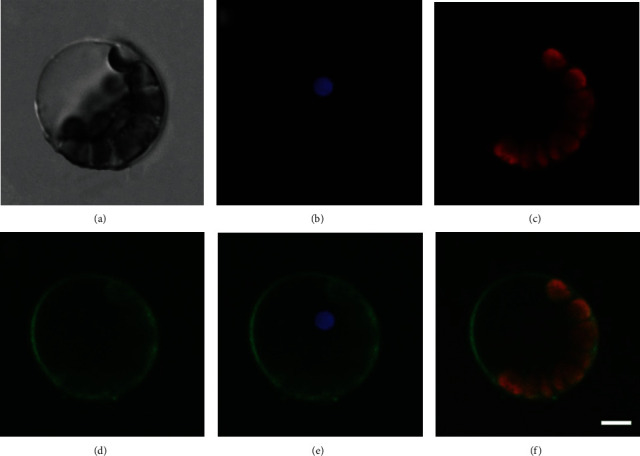
Subcellular localization of LpAGPL1. (a) bright, (b) DAPI, (c) acridine orange (AO), (d) GFP, (e) GFP + DAPI merge, and (f) GFP + AO merge. Bar = 10 *μ*m.

Analysis of LpAGPs subcellular localization was performed to determine if they are plastid or cytoplasmic proteins. The five *LpAGP* coding regions were cloned into the vector 16318-hGFP, which was then transformed into *A. thaliana* protoplasts. Fluorescence images of the protoplasts showing the GFP expression were taken using a confocal microscope. The results demonstrated that the five AGPase subunits of *L. punctata* are uniformly located in the chloroplast, indicating that they are plastid proteins (Figure 6, Figure S1-S4). Notably, the AGPase LSs are possibly located on the cell membrane (Figure S3, S4).

## 4. Discussion

Duckweed is a regenerable bioenergy crop with enormous potential for boosting energy supplies. Its bioethanol yield is about 50% higher than maize, demonstrating a promising starch source for bioethanol production [29]. The genomic characteristics of two *Spirodela* and *Lemna* species have been reported [30, 31]. Although less studied, *L. punctata* can quickly accumulate high starch levels under nutrient starvation or uniconazole [11, 32]. However, the mechanism of its starch accumulation remains unknown. Therefore, studying how *L. punctata* differs from other species is essential in utilizing its full potential.

Wang et al. [31] reported the first genome of *Lemnaceae*: *S. polyrhiza* in 2014. Based on their results, *S. polyrhiza* genome has 158 Mb and encodes 19,623 proteins, including four AGPase, one GBSS, four soluble starch synthase (SSS), and three starch branching enzyme (SBE) coding genes. In comparison to *S. polyrhiza*, *L. punctata* has five AGPase, two GBSS, two SSS, and five SBE coding genes. The three genes encoding AGPase large subunits of *S. polyrhiza* have been cloned; however, the small subunit coding gene is yet to be cloned. A comparison between the three AGPase LSs of *L. punctata* and the AGPase of *S. polyrhiza* shows that the similarity between LpAGPL1 and SpAGPL1 (Genbank AEV40471), LpAGPL2 and SpAGPL2 (AEV40472), and LpAGPL3 and SpAGPL3 (AEV40474) is 94.39%, 95.52%, and 83.30%, respectively. These results suggest that the LpAGPSs and SpAGPSs coding genes may play similar functions. Unlike *S. polyrhiza*, which develops a seed-like turion for storing starch [33], *L. punctata* has a different storage mechanism. Metabolic pathway analysis showed that nutrient starvation and uniconazole treatment enhance starch anabolism but weaken starch catabolism in *L. punctata*, resulting in high starch accumulation by modulating the global carbon metabolism flux [34].

Full-length *LpAGPS1* and *LpAGPL1* cDNA were previously cloned from *L. punctata* strains 0202 [35] and 5632 [36]. The lengths of *LpAGPS1* (1,578 bp) and *LpAGPL1* (1,554 bp) cDNA were the same in the two *L. punctata* strains. In contrast, the corresponding sequences cloned in 2015 (*LeAPS*: KJ603243.1; *LeAPL1*: KJ603244.1) by Taq DNA polymerase are different from the sequences cloned in this study [35]. Here, we cloned all the five *LpAGP* cDNAs. Phylogenetic analyses showed that three LpAGPLs (LpAGPL1, LpAGPL2, and LpAGPL3) are more conserved than two LpAGPSs (LpAGPS1, LpAGPS2). Notably, LpAGPS2 has a rather distant phylogenetic relationship with the other four LpAGPs. The sequence similarity between LpAGPS2 and the AGPase small subunit of *Theobroma cacao* was 85.13%. Furthermore, we found that LpAGPS2 has a high degree of sequence homology to many GDP-mannose pyrophosphorylase (GMPPA, EC: 2.7.7.13), including the GMPPA of *Capsicum annuum* (86% similarity). The 7-386 region of LpAGPS2 has a glgC domain like other AGPases (PRK05293, *E* value =6.69 × *e*^−12^). Of note, studies have shown that the catalytic substrates of AGPase and GMPPA are alpha-D-glucose-1-phosphate (Glc1P) and alpha-D-mannose-1-phosphate, respectively [37, 38]. These two enzymes catalyze similar reaction types, considering that glucose is an epimer of mannose. It is hypothesized that the LpAGPS2 protein may have both AGPase and GMPPA enzyme activities; however, further studies are needed to verify this.

Starch is a stored polysaccharide in plants. Thus, studies have attempted to maximize starch content in *L. punctata* for several years. Some studies have shown that nitrogen-limited cultivation [39], uniconazole [40, 41], abscisic acid (ABA) [42], nutrient starvation [11], and pectinase [43] can stimulate high starch accumulation in duckweed. For example, the starch levels of *S. oligorrhiza* increased to 75% DW [44] when cultivated in a glucose medium without phosphate. This is the highest starch accumulation in duckweed ever recorded. Regarding *L. punctata*, 52.9% is the highest ever recorded DW starch content [10]. Studies have shown that enzyme activities related to starch biosynthesis increase during the starch accumulation process, especially the activity of AGPase [41]. However, not all AGPase subunit combinations can form active AGPase in plants. AGPase SSs or LSs solely have no catalytical and allosteric properties without subunit synergy [45]. However, there are exceptions; for example, the tomato L3 subunit has catalytic activity as a monomer [46].

In this study, yeast two-hybrid and bacterial coexpression assays were performed to detect the interaction of different subunits. The results showed that LpAGPS1 failed to interact with LpAGPL1 but was weakly associated with LpAGPL2. Additionally, LpAGPS1 interacts with LpAGPL3 to form an active AGPase. LpAGPS2 interacts with all the LpAGPLs; its coexpression in *E. coli* caused a color change when exposed to iodine staining, indicating that it can interact with LpAGPLs despite being different from most AGPase SSs. Meanwhile, BiFC assay showed that LpAGPS1 interacts with LpAGPL2. This could be because LpAGPS1 and LpAGPL2 can form a functional AGPase with weak activity.

AGPase is typically located in the plastids of most plant tissues. However, most AGPases in mid-developmental stages of rice, maize, barley endosperm, and early-stage wheat endosperm are localized in the cytoplasm [21, 26, 47–51]. Two AGPase classes have been reported in plants, including cytosolic AGPase and plastidial AGPase. Correspondingly, AGPase subunits are categorized into small cytosolic, small plastidial, large cytosolic, and large plastidial subunits. In this study, five *L. punctata* AGPase subunits were fusion-expressed with hGFP in *Arabidopsis* protoplasts. The results revealed that the green fluorescence of the five fusion-expressed AGPase subunits are all located in chloroplast, suggesting that the five AGPase subunits are all plastidial subunits. In cereals, a significant portion of AGPase activity occurs in the cytosol of endosperms and a minor portion in amyloplasts [47]. In contrast, AGPases of most dicotyledonous plants are plastid proteins. Spatiotemporal expression of AGPases results in different AGPase compositions in various tissues, adapting to specific metabolic requirements of specific tissues.

During starch biosynthesis, various enzymes jointly determine the quality and quantity of starch. Therefore, plant breeders hope to improve starch synthesis and yield by increasing the expression of starch synthesis pathway-related enzymes, in which AGPase is considered important [52]. As a fast-growing aquatic plant, duckweed can adapt to the various pH and temperature ranges [53]. Duckweed is used for industrial production of ethanol and enzymes [54] and used as a bioreactor for producing antibodies, vaccines, etc. due to its relatively lower production cost [55, 56]. However, most have studies focused on the industrial application of *Spirodela, Lemna,* and *Wolffia* belonging to Lemnaaceae, while studies on *Landoltia* are rarely reported. Thus, this study lays a theoretical foundation on the application of *Landoltia* in industries, medicine, and other fields.

## 5. Conclusion

In this study, five *LpAGPs* of *L. punctata* (duckweed) were cloned and characterized. Nutrient starvation significantly upregulated the expression of *LpAGPS1*, *LpAGPL2*, and *LpAGPL3*. The yeast two-hybrid and BiFC assays revealed the interactions between LpAGPS1/LpAGPL2, LpAGPS1/LpAGPL3, LpAGPS2/LpAGPL1, LpAGPS2/LpAGPL2, and LpAGPS2/LpAGPL3. All the five LpAGPs are localized in the chloroplast. These results pave the way for future functional tests of *LpAGPs* in vivo.

## Figures and Tables

**Table 1:** Alignment analysis of large and small subunits of AGPase in *L. punctata.*

Protein	Length (aa)	Transit peptide (aa)	Sequence alignment		
			Protein name	Genbank accession	Identity
LpAGPS1	525	67	PfAGPS	AAF66434.1	87.95%
LpAGPS2	417	17	TcAGPS	EOY20587.1	85.61%
LpAGPL1	517	22	SpAGPL1	AEV40471	94.39%
LpAGPL2	536	78	SpAGPL2	AEV40472	95.52%
LpAGPL3	515	36	SpAGPL3	AEV40474	83.30%

## Data Availability

Sequences were deposited in the National Center for Biotechnology Information (NCBI) (https://www.ncbi.nlm.nih.gov/).

## Abbreviations

AGPase: ADP-glucose pyrophosphorylase
LS: Large subunit
SS: Small subunit
DW: Dry weight
BiFC: Bimolecular fluorescence complementation
IPTG: Isopropyl *β*-D-thiogalactoside
DAPI: 4′,6-Diamidino-2-phenylindole.

## Supplementary Materials

Supplementary 1 Figure S1: subcellular localization of LpAGPS1.

Supplementary 2 Figure S2: subcellular localization of LpAGPS2.

Supplementary 3 Figure S3: subcellular localization of LpAGPL2.

Supplementary 4 Figure S4: subcellular localization of LpAGPL3.

Supplementary 5 Table S1: primers for yeast two-hybrid, coexpression of bacteria, subcellular localization, and bimolecular fluorescence complementation.
