# Supplementary figures and images for: Characterization of Various Subunit Combinations of ADP-Glucose Pyrophosphorylase in Duckweed (Landoltia punctata)
Source: Biomed Res Int. 2022 Mar 9;2022:5455593. doi: 10.1155/2022/5455593 (PMC8927976; doi:10.1155/2022/5455593)

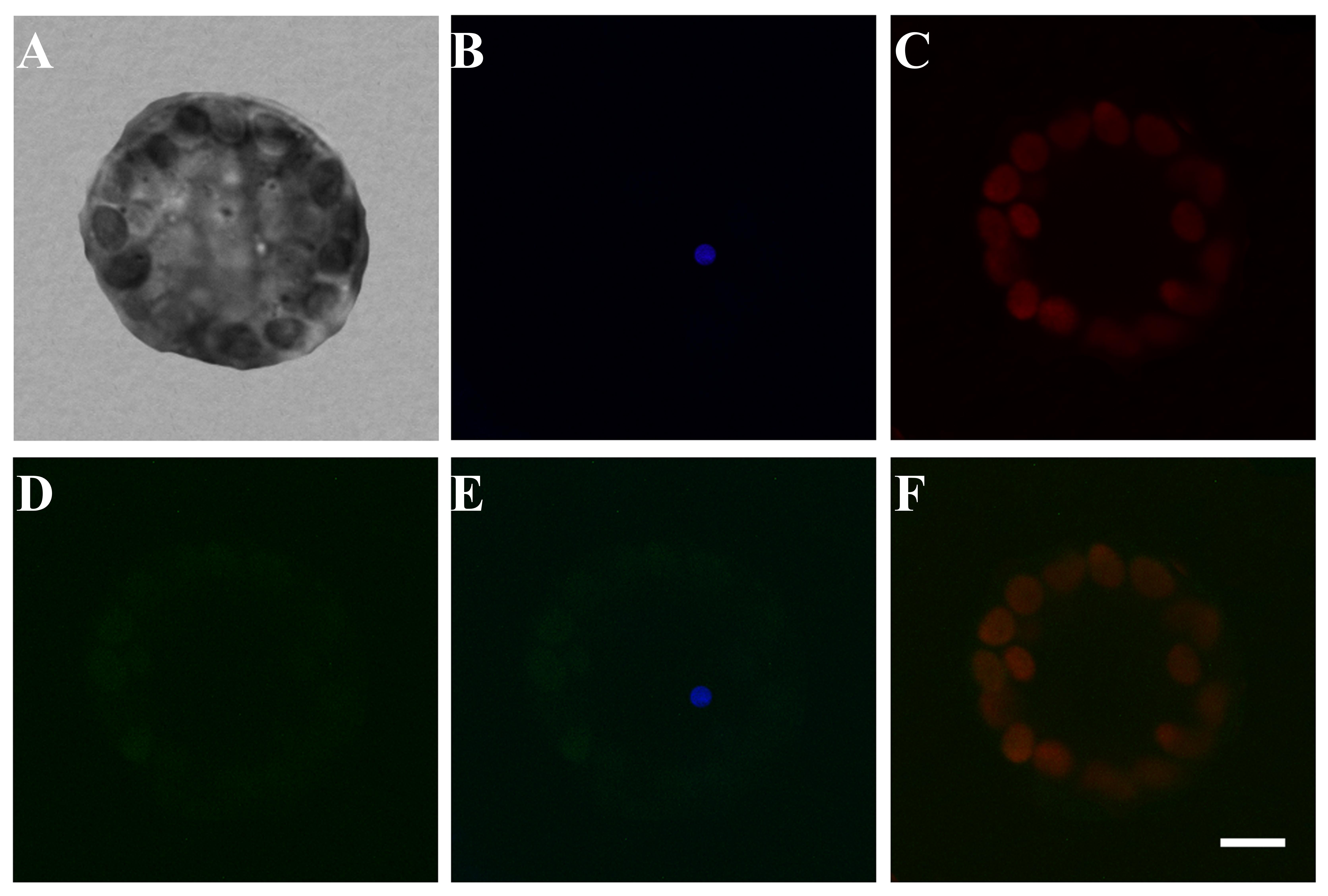

Supplement: Supplementary 1 — Figure S1: subcellular localization of LpAGPS1. [file 5455593.f1.jpg]

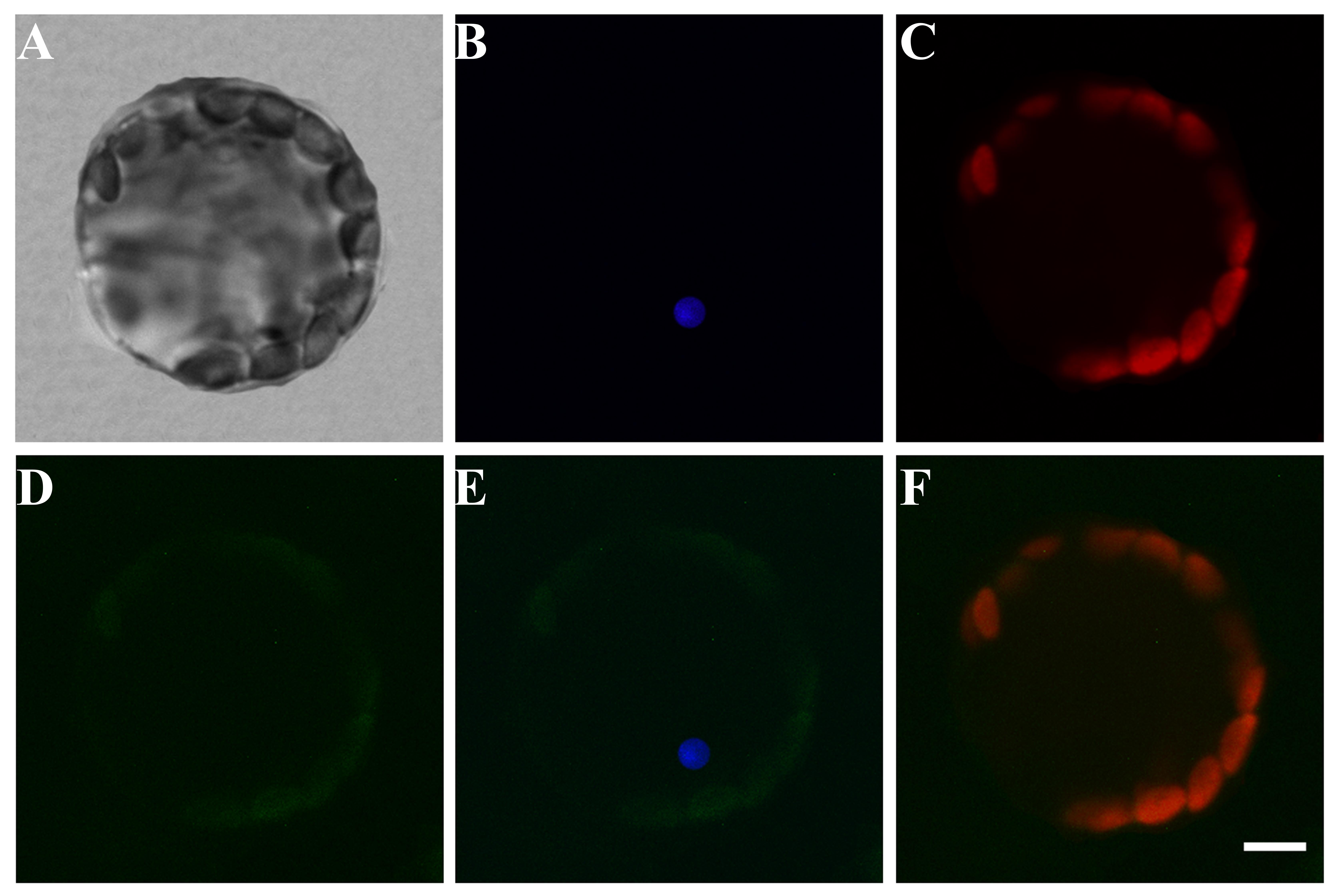

Supplement: Supplementary 2 — Figure S2: subcellular localization of LpAGPS2. [file 5455593.f2.jpg]

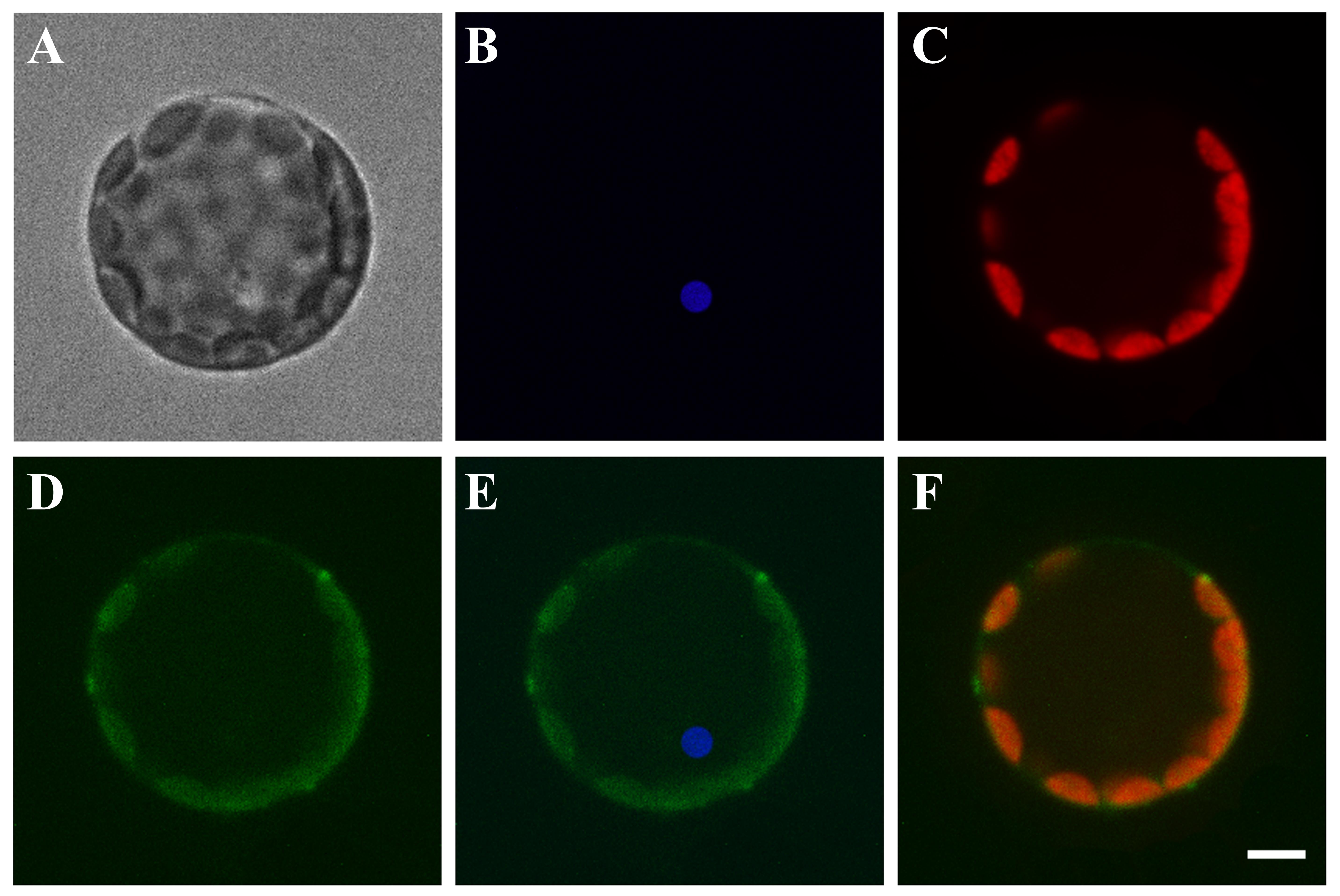

Supplement: Supplementary 3 — Figure S3: subcellular localization of LpAGPL2. [file 5455593.f3.jpg]

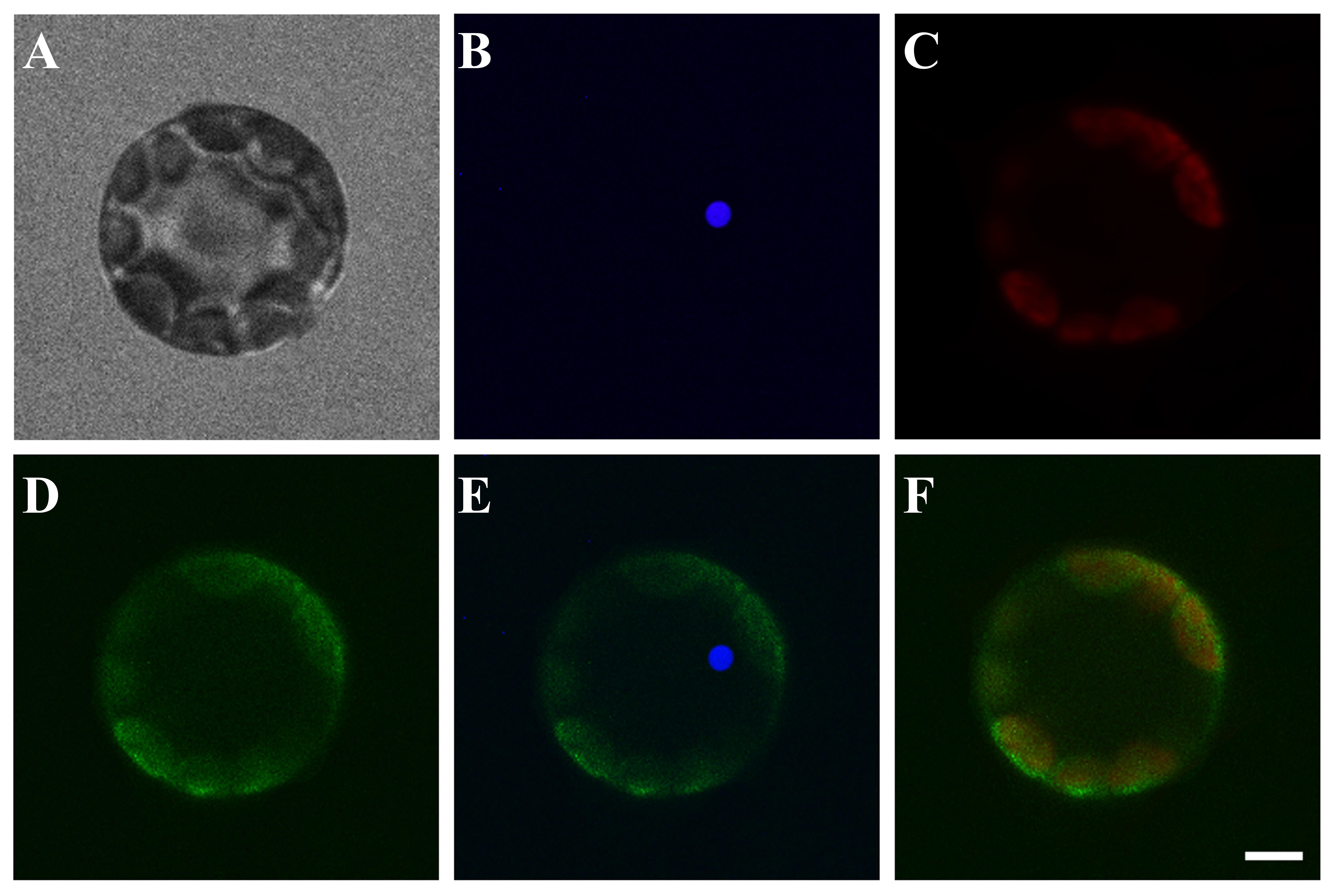

Supplement: Supplementary 4 — Figure S4: subcellular localization of LpAGPL3. [file 5455593.f4.jpg]
