# Supplementary material for: Characterization of Various Subunit Combinations of ADP-Glucose Pyrophosphorylase in Duckweed (Landoltia punctata)
Source: Biomed Res Int. 2022 Mar 9;2022:5455593. doi: 10.1155/2022/5455593 (PMC8927976; doi:10.1155/2022/5455593)
Supplement: Supplementary 5 — Table S1: primers for yeast two-hybrid, coexpression of bacteria, subcellular localization, and bimolecular fluorescence complementation. [file 5455593.f5.pdf]

Table S1 Primers for yeast two-hybrid, co-expression of bacteria, subcellular localization and BiFC

	Primer name	Primer sequence (5'-3')
**Yeast two-hybrid**	S1F1	AGCTCCATGGACGCGCCGATCGTCGTCTCCCCT
	S1R1	ATCGGGATCCTCATATGATGGTTCCGCTAGGGATC
	S2F1	AGCTCCATGGGGAGTTCACAGGAGAAGGTGGT
	S2R1	ATCGGGATCCTCAGAGGATGATCTCTTCCTGTAC
	L1F1	AGCTACATGTACTGCTTCCGACGAGGGGAGATAATG
	L1R1	ATGCGAATTCTCATATGACAAGGCCGTCCTTTA
	L2F1	AGCTACATGTACGCCGATATCCCCAAGGATC
	L2R1	ATGCGAATTCTCAGATGACAGTGCCGTCGGGAAT
	L3F1	AGCTACATGTACCAAGTGCACGGACGCGGC
	L3R1	ATGCGAATTCTCAGATAACGGTGCCGTCCTTGA
**Co-expression**	S1F2	AGCTCCATGGACGCGCCGATCGTCGTCTCCCCT
	S1R2	ATCGGGATCCTCATATGATGGTTCCGCTAGGGATC
	S2F2	AGCTCCATGGGGAGTTCACAGGAGAAGGTGGT
	S2R2	ATCGGGATCCTCAGAGGATGATCTCTTCCTGTAC
	L1F2	ATGCGAATTCACTGCTTCCGACGAGGGGAGATAATG
	L1R2	AGCTAAGCTTTCATATGACAAGGCCGTCCTTTA
	L2F2	ATGCGAATTCACGCCGATATCCCCAAGGATC
	L2R2	AGCTAAGCTTTCAGATGACAGTGCCGTCGGGAAT
	L3F2	ATGCGAATTCACCAAGTGCACGGACGCGGC
	L3R2	AGCTAAGCTTTCAGATAACGGTGCCGTCCTTGA
**Subcellular localization**	S1F3	GCGTCGACATGGCGCCGATCGTCGTCTC
	S1R3	CGGGATCCTATGATGGTTCCGCTAGGGATC
	S2F3	GCGTCGACATGGGGAGTTCACAGGAGAAG
	S2R3	CGGGATCCGAGGATGATCTCTTCCTGTAC
	L1F3	GCGTCGACATGTGCTTCCGACGAGGGGAG
	L1R3	CGGGATCCTATGACAAGGCCGTCCTTTATC
	L2F3	GCGTCGACATGGCCGATATCCCCAAGGAT
	L2R3	CGGGATCCGATGACAGTGCCGTCGGGAATG
	L3F3	CCGCTCGAGATGCAAGTGCACGGACGCGGCGGCCGG
	L3R3	CGGGATCCCTTGCAATTGTCCATCTTGGTGGGC
**Bimolecular fluorescence complementation**	S1F4	TGGCGCGCGCGCCGATCGTCGTCTCCCCT
	S1R4	GACAGTACTATCGATGTATGATGGTTCCGCTAGGGATC
	S2F4	TGGCGCGCCACTAGTGGAATGGGGAGTTCACAGGAGAAGG
	S2R4	GACAGTACTATCGATGGAGGATGATCTCTTCCTGTACGC
	L1F4	TGGCGCGCTGCTTCCGACGAGGGGAGATAATG
	L1R4	GACAGTACTATCGATGTATGACAAGGCCGTCCTTTATCTC
	L2F4	TGGCGCGCGCCGATATCCCCAAGGATC
	L2R4	GACAGTACTATCGATGGATGACAGTGCCGTCGGGAATG
	L3F4	TGGCGCGCCAAGTGCACGGACGCGGC
	L3R4	GACAGTACTATCGATGGATAACGGTGCCGTCCTTGATGG
